# Prevalence of medication-related falls in 200 consecutive elderly patients with hip fractures: a cross-sectional study

**DOI:** 10.1186/s12877-020-01532-9

**Published:** 2020-03-30

**Authors:** Charlotte Uggerhøj Andersen, Pernille Overgaard Lassen, Hussain Qassim Usman, Nadja Albertsen, Lars Peter Nielsen, Stig Andersen

**Affiliations:** 1 Department of Clinical Pharmacology, Aalborg University Hospital, Mølleparkvej 8, 9000 Aalborg, Denmark; 2 Department of Clinical Medicine, Aalborg University, Aalborg, Denmark; 3 Department of Clinical Pharmacology, Aarhus University Hospital, Aarhus, Denmark; 4 Department of Geriatric and Internal Medicine, Aalborg University Hospital, Aalborg, Denmark

**Keywords:** Fall-risk-increasing drugs, Potentially inappropriate medication, Fall, Polypharmacy, Geriatrics

## Abstract

**Background:**

Hip fractures constitute a major health problem in elderly people and are often fall-related. Several factors can contribute to a fall episode leading to hip fracture, including fall-risk-increasing drugs (FRIDs), which are often used by elderly people.

We aimed to investigate the prevalence of medication-related falls and to assess the role of FRIDs and potentially inappropriate medications (PIMs) in a population of elderly patients hospitalized for a hip fracture.

**Methods:**

We reviewed the patient records of 200 consecutive patients, aged ≥65 years, who were admitted for a hip fracture and evaluated whether medications were likely to have contributed to the fall episode. PIMs were identified using the Screening Tool of Older Persons’ Prescriptions version 2 (STOPP) and by evaluating indications, contra-indications and interactions of the prescribed medications for each patient.

**Results:**

FRIDs were used by 175 patients (87.5%). Medications were considered a likely contributor to the fall in 82 patients (41%). These were most often psychotropic medications alone or in combination with antihypertensives and/or diuretics. The 82 patients with suspected medication-related falls used more medications, FRIDs and PIMs than the rest of the patients, and in 74 (90%) of the 82 patients, at least one medication considered to be a contributor to the fall was also a PIM.

**Conclusions:**

The prevalence of suspected medication-related falls was 41%. It seems likely that a medication review could have reduced, though not eliminated, the risk of falling in this group of patients.

## Background

A hip fracture is associated with considerable socio-economic costs [1] and constitutes a high-risk situation for an elderly patient, as the mortality for patients older than 65 years is 12–35% within the first year after the fracture [2] and remains elevated for several years [3]. Several medications have been identified as fall-risk-increasing drugs (FRIDs) [4–8]. The association between an increased risk of falling and the use of psychotropic medications, such as antidepressants, antipsychotics and benzodiazepines, seems well established, as indicated by odds ratios ranging from 1.3 to 2 in a recent metanalysis [8]. The association between falls and the use of cardiovascular medications, including antihypertensives and antiarrhythmics, does not seem quite as consistent [6]. However, in clinical practice, cardiovascular medications are often regarded as FRIDs, [9, 10] as their adverse effects can directly or indirectly cause dizziness, hypotension and orthostatic hypotension. Several reports have shown that more than 90% of elderly people experiencing a fall or a hip fracture are taking FRIDs [10, 11]. Thus, FRIDs can be regarded as a modifiable risk factor. Furthermore, a recent study showed that 85% of elderly patients with a hip fracture were prescribed potentially inappropriate medications (PIMs), which may be unnecessary or entail a high risk of adverse effects [12]. The Screening Tool of Older Persons’ Prescriptions (STOPP) [13] may guide the performance of a medication review, both by pointing out situations in which certain medications are potentially inappropriate and by identifying certain FRIDs as PIMs.

Given the high prevalence of FRIDs and PIMs among elderly patients, it may be hypothesized that medication reviews and interventions to reduce FRIDs and PIMs in this group would effectively reduce the risk of falling. However, two recent randomized studies investigating the effect of FRID-withdrawal among more than 600 elderly people experiencing a fall [9], and the effect of medication reviews [14] among 199 elderly patients with hip fractures [14] did not find an effect of these interventions on the rate of falls during a 12-month follow-up period. Possible explanations include competing risk factors for falling, such as comorbidities, and impaired cognition, balance, or vision. In addition, extrinsic factors influencing the subject [15, 16] may play a role in many falls.

Even though the prevalence of FRID users among patients with a hip fracture is high, it is not known how often FRIDs actually contribute to the fall. This knowledge is essential to understanding how much the incidence of hip fracture could potentially be reduced by stopping the use of these medications. Thus, the aims of our study were to estimate the prevalence of medication-related falls leading to a hip fracture in a population of elderly patients admitted to a joint orthopaedic and geriatric ward and to assess the role of FRIDs and PIMs.

## Methods

### Study design and population

This study was a retrospective cross-sectional study. Two hundred consecutive patients with hip fracture, aged 65 years or older, admitted to a Danish University Hospital during a period of 24 weeks in 2017 were identified by a search in the hospital’s database using the ICD-10 codes for fracture of the femur (DS72-DS729).

### Evaluation and definition of medication-related falls, FRIDs and PIMs

A consultant in Clinical Pharmacology (CUA) reviewed all patient records, focusing on 1) the description of the fall episode, including fall-related symptoms and the conclusions regarding the causes of the fall, made by the attending geriatrician during admission; 2) comorbidities, demographic data and medication list at the time of admission; 3) blood pressure, respiratory frequency, peripheral saturation, body temperature and heart rate at the time of and during admission; 4) laboratory data including c-reactive protein, leucocyte count, electrolytes, haemoglobin, liver and renal parameters, and blood glucose; 5) the results of other diagnostic evaluations performed during admission; and 6) medication withdrawals or changes during admission.

FRIDs were defined in accordance with previous work on fall-risk-related medications [6–8, 13, 17, 18]: 1) psychotropic medications (antidepressants, antipsychotics, antiepileptic medications, medications for Parkinson’s disease, medications for dementia, first-generation antihistamines, benzodiazepines or benzodiazepine-like medications (zopiclone and zolpidem) and opioids); 2) cardiovascular medications (calcium antagonists, angiotensin converting enzyme (ACE)-inhibitors, angiotensin-II receptor (AT-II) antagonists, beta-adrenoceptor antagonists, alpha-adrenoceptor antagonists, diuretics, nitrous vasodilators, and anti-arrhythmic medications); and 3) urinary antispasmodics. PIMs were identified by review of the patients’ medication lists according to the STOPP version 2, [13] available from the Danish Geriatric Society. Furthermore, the indications, contra-indications and interactions for each prescribed medication, as listed in the Summary of Product Characteristics found on the homepage of the Danish Medicines Agency, were also considered for each patient. All data mentioned above was entered in case report forms during the initial review.

The clinical pharmacologist excluded a suspected medication-related fall if a patient had not experienced a fall, was not using any medications or FRIDs, or if a non-medication-related fall cause was described in the patient record. Otherwise, the clinical pharmacologist and a consultant in Geriatrics (POL, HU, or NA) discussed the case in order to obtain a consensus about whether one or more medications were likely contributors to the fall. Medications were generally considered likely contributors to the fall if their effects, adverse effects or interactions could have caused or aggravated symptoms or clinical findings related to the fall episode, for example, orthostatic hypotension. If we concluded that medications were likely contributors to the fall, we defined the patient as having had a suspected medication-related fall. If the fall was more likely explained by the consequences of acute or chronic disease, tripping or extrinsic factors, we did not consider medications likely contributors. Patients who were not attended by an on-site geriatrician during admission were evaluated retrospectively following the same procedure as that used for the rest of the patients.

### Data handling and statistical analysis

Study data were collected and managed using REDCap (Vanderbilt, USA) electronic data capture tools hosted at Aalborg University. REDCap is a secure, web-based application designed to support data capture for research studies [19]. Data were exported for statistical analysis or graphics in STATA (StataCorp. 2017. Stata Statistical Software: Release 15. College Station, TX: StataCorp LLC). The distribution of variables was evaluated according to histograms and Q-norm plots, and data that had a normal distribution were summarized as means ± standard deviations (SDs). Variables that were not normally distributed were summarized by medians [25th percentile, 75th percentile], and differences between two groups were tested by the Wilcoxon rank-sum test. Differences in the prevalence of diseases between patients with and without a suspected medication-related fall were analysed by two-sample z-test. Differences in age, clinical data and the number of medications and FRIDs among multiple groups were analysed by regression with a bootstrap analysis. The differences between proportions of patients with PIMs among multiple groups were compared by pairwise two-sample z-tests. Power calculation to estimate study size was not performed due to lack of data to support this. Analyses were performed without imputation of missing data. The number of patients with missing data are indicated for each variable in footnotes of tables. Percentages of patients with a given condition (e.g. sodium < 132 mmol/l) were calculated by dividing the number of confirmed cases with the total number of patients in the population (200).

## Results

### Prevalence of suspected medication-related falls

**Fig. 1:**
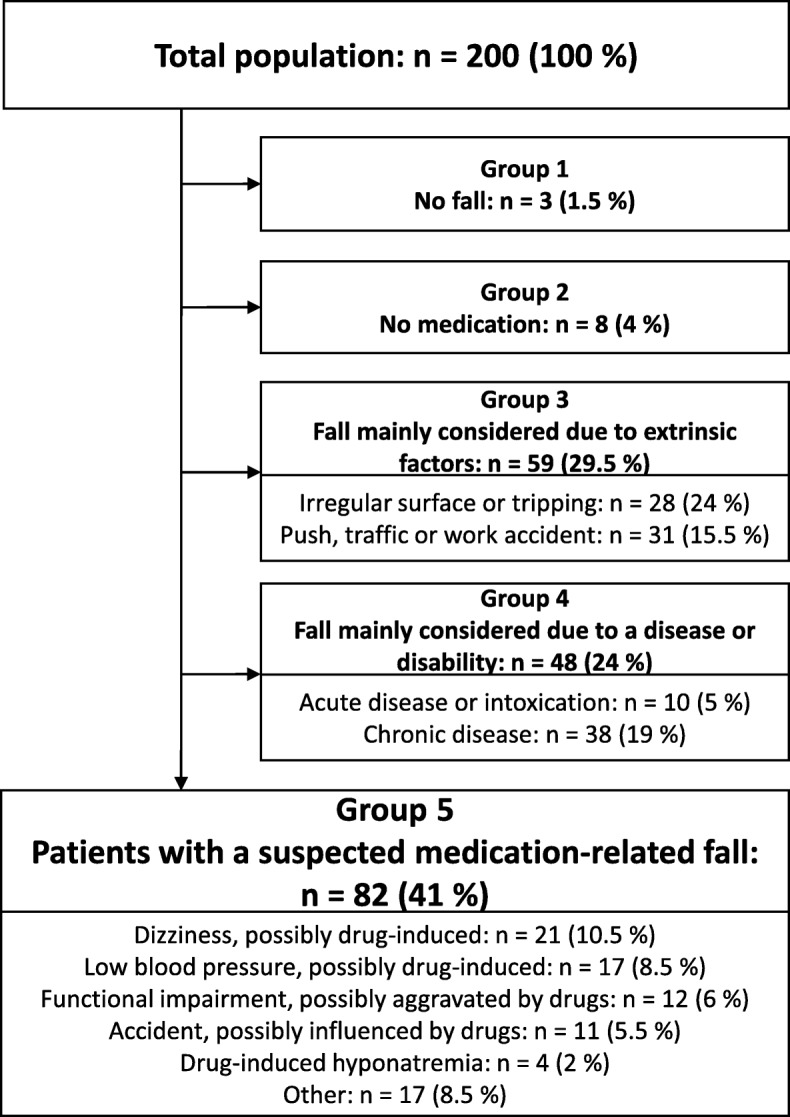
Main causes of falls. Figure 1. Flow diagram showing the selection procedure for the patients having a medication-related fall, as evaluated with the joint expertise of a clinical pharmacologist and a geriatrician

A fall preceded the hip fracture in 197 (98.5%) patients, and the fall was considered a low energy trauma, indicating an osteoporotic fracture, in 175 (87.5%) patients. Three patients (1.5%) (group 1) did not experience a fall, and eight (4%) (group 2) did not take any medication. In 59 patients (29.5%) (group 3), the fall episode seemed more likely to have been caused by an extrinsic factor, such as a push by a large animal or another person, or tripping. Consequences of a chronic or acute disease, such as influenza, urinary tract infection or uncontrolled atrial fibrillation, were considered the main cause of the fall in 48 patients (24%) (group 4). Finally, we considered 82 (41%) patients as having had a suspected medication-related fall (group 5) (Fig. 1). The most frequent reason for medications to be regarded as a contributor to the fall was dizziness in patients using medications with dizziness as a known adverse effect (*n* = 21 (10.5%)), followed by the presence of low blood pressure or symptoms of orthostatic hypotension in patients who were using medications able to decrease blood pressure (*n* = 17 (8.5%)). Medications were suspected to contribute to a fall by impairing the functional level in 12 patients (6%) and to contribute to accidental happenings leading to a fall by influencing cognition or balance in 11 patients (5.5%). In both instances, psychotropic drugs were the most commonly involved. Diuretics were thought to play a role by inducing hyponatremia in four patients (2%). Finally, the circumstances of the fall episode were not well described in 17 patients (8.5%), but the presence of FRIDs and no other obvious causes of the fall led us to define these as having had a suspected medication-related fall.

A consultant geriatrician had performed an onsite evaluation of 170 (85%) of the patients in the study population during admission.

### Demographics, comorbidities and use of medication in the total population

A summary of the populations’ demographic and clinical characteristics is shown in Table 1.

**Table 1:** Characteristics of the study population

General characteristics	
Female (*n* (%))	136 (68)
Age (years)	82 [76,88]
Age > 80 years (*n* (%))	115 (57.5)
Height (cm)	166 ± 9 cm
Weight (kg)	67 ± 14 kg
Body mass index (kg/m^2^)	24 ± 4
Body mass index < 18.5 (*n* (%))	17 (8.5)
**Residence (*n*(%))**	
Private home	158 (79)
Nursing home	35 (17.5)
Other	9 (4.5)
**Type of fall and fracture (*n*(%))**	
Low-energy trauma defining an osteoporotic fracture	175 (87.5)
Not low-energy trauma	22 (11)
No fall	3 (1.5)
**Clinical findings (*n*(%))**	
First SBP at admission < 120 mmHg	12 (6)
Lowest measured SBP during the admission < 120 mmHg	135 (67.5)
First PS at admission < 90%	22 (11)
First RF at admission > 20 /min	23 (11.5)
First TP > 38.0 °C	11 (5.5)
**Abnormal laboratory findings at the time of admission (*n*(%))**	
Estimated glomerular filtration rate^*^ < 60 ml/min	74 (37)
Estimated glomerular filtration rate^*^ < 30 ml/min	13 (6.5)
C-reactive protein > 100 mg/l	12 (6)
Haemoglobin < 6 mmol/l	11 (5.5)
Sodium < 132 mmol/l	12 (6)
Potassium < 3.2 mmol/l	5 (2.5)
Thyroid stimulating hormone < 0.3 m IU/l or > 4.5 mIU/l	20 (10)
Alanine amino transferase > 50 U/l in men or > 35 U/l in women	12 (6)
Glucose < 4 mmol/l	0 (0)
Glucose > 10 mmol/l	14 (7)
Albumin < 34 g/l	69 (34.5)

Table 1. Detailed description of the characteristics of the study population. Abbreviations: *CNS* central nervous system *SBP* systolic blood pressure, *PS* peripheral saturation, *RF* respiratory frequency, *TP* body temperature. ^*^: calculated by the EPI-CKD formula. Number of patients with missing data for each variable: age: 0, height: 10, weight: 17, body mass index: 24, first SBP: 2, lowest SBP: 8, PS: 4, RF: 4, TP: 8, Estimated glomerular filtration rate: 1, c-reactive protein: 9, Haemoglobin: 3, sodium: 2, potassium: 2, thyroid stimulating hormone: 37, alanine amino transferase: 8, glucose: 16, albumin: 2. Percentages of patients with a given condition (e.g. sodium < 132 mmol/l) were calculated by dividing the number of confirmed cases with the total number of patients in the population (200)

One or more comorbidities were present in 195 (97.5%) patients, and the most common comorbidities were hypertension, osteoporosis and atrial fibrillation (Table 2).

**Table 2:** The prevalence of comorbidities at the time of admission occurring in 5% or more of patients

	Whole population	Patients without a medication-related fall	Patients with a medication-related fall	*P* value (patients with vs. without a medication-related fall)
*n* (%)	200 (100)	118 (59)	82 (41)	
**Prevalence of comorbidity (*n*(%))**				
Hypertension	90 (45)	49 (42)	41 (50)	0.2
Osteoporosis	46 (23)	28 (24)	18 (22)	0.8
Atrial fibrillation	44 (22)	21 (18)	23 (28)	0.09
Previous ischaemic stroke	36 (18)	21 (18)	14 (17)	0.9
Chronic obstructive pulmonary disease	32 (16)	16 (13)	16 (19)	0.2
Dementia	30 (15)	15 (13)	15 (18)	0.3
Previous fracture (hip or spine)	29 (14.5)	12 (10)	17 (20)	0.04
Ischaemic heart disease	27 (13.5)	11 (9)	20 (24)	0.04
Type 2 diabetes	27 (13.5)	19 (14)	8 (10)	0.4
Chronic renal failure	25 (12.5)	16 (13)	9 (11)	0.6
Depression	22 (11)	9 (7)	13 (16)	0.07
Cancer	18 (9)	12 (10)	6 (7)	0.5
Chronic heart failure	14 (7)	10 (8)	4 (5)	0.3
Visual or hearing impairment	14 (7)	13 (7)	10 (7)	0.9
Hyperthyreosis	12 (6)	7 (6)	5 (6)	1
Arthrosis	12 (6)	6 (5)	5 (6)	0.7
Hypothyreosis	10 (5)	6 (5)	4 (5)	1
Chronic alcoholism	10 (5)	5 (4)	4 (5)	1

Table 2. Comorbidities at the time of admission with a prevalence of 5 % or higher. One patient could have several diagnoses. Differences between patients with and without a medication-related fall were tested by the two-sample z-test

The median number of prescribed medications used at admission was seven, ranging from 0 to 27. At least 1 FRID was prescribed to 175 (87.5%) patients, and the median number of used FRIDs was three, ranging from zero to seven. A detailed list of medications used at the time of admission is available in Table 1S in the on-line supplementary material.

### Characteristics of patients with a suspected medication-related fall

The group of patients with a suspected medication-related fall was markedly older than the group in which an extrinsic factor or tripping was considered the most likely reason (group 3). Otherwise, only the number of prescribed medications and FRIDs at the time of admission and the proportion of PIMs were significantly higher in patients with a suspected medication-related fall than in patients in the other groups (Table 3). Regarding comorbidities, patients with a suspected medication-related fall had a higher prevalence of osteoporosis and ischaemic heart disease when compared to the rest of the patients (Table 2).

**Table 3:** Comparison of demographics, clinical parameters and use of medications for the groups 1 to 5 shown in Fig. 1

	Group no.					
	1	2	3	4	5	*p*-value (regression with bootstrap)
*n* (%)	3 (1.5)	8 (4)	59 (29.5)	48 (24)	82 (41)	
Description	No fall	No medication	Extrinsic factor or tripping	Disease or disability	Medication-related fall	
Age (years)	78 [77–84]	72 [70–77.5]	79 [73–85]*#	83 [78.5–89]	84 [77–89]	0.0001
Body mass index (kg/m^2^)	25 ± 5	25 ± 4	25 ± 4	24 ± 4	24 ± 4	0.7
SBP at admission (mmHg)	182 ± 27	150 ± 17	150 ± 25	160 ± 22	150 ± 27	0.06
Lowest SBP measured during admission (mmHg)	107 ± 33	116 ± 17	111 ± 23	111 ± 20	109 ± 20	0.8
PS at admission (%)	94 ± 4	97 ± 3	94 ± 5	94 ± 4	94 ± 4	0.1
TP at admission	37.4 ± 0.7	36.6 ± 0.7	36.8 ± 0.5#	37.1 ± 0.8	36.9 ± 0.6	0.04
Estimated glomerular filtration rate (ml/min)	51 [6–61]	81.5 [73–88] *	71 [50–86]	75 [47–86]	66 [44–82]	0.04
Sodium (mmol/l)	140 [137–143]	139 [136–141]	140 [138–141]*#	137 [135–141]	138 [136–141]	0.002
Number of drugs at admission	11.6 ± 5.1	–	6.4 ± 4.3*	6.4 ± 3.3*	9.7 ± 4.0	< 0.0001
Number of FRIDS at admission	2.3 ± 1.2	–	2.3 ± 1.4*	1.75 ± 1.3*	3.7 ± 1.2	< 0.0001
Proportion of patients with PIMs (n (%))	3 (100)	–	34 (57) Ɨ	25 (52) Ɨ	79 (96)	–

The table shows means ± standard deviations for parametric data and medians [interquartile range] for nonparametric data. Differences in the numeric data between groups were analysed by regression with a bootstrap analysis; *: *p*-value < 0.05 vs group 5. #: *p*-value < 0.05 vs group 4. Differences in the proportion of patients with PIMs were analysed by pairwise two-sample z-tests among groups 3, 4, and 5; Ɨ: *p*-value < 0.001 vs group 5. Abbreviations: *SBP* systolic blood pressure, *PS* peripheral saturation, *TP* temperature, *FRIDS* Fall-risk-increasing medications, *PIMs* Potentially inappropriate medications. Number of persons with missing data for each variable: age: 0, body mass index: 24 (0, 1, 6, 6, and 11 in group 1, 2, 3, 4, and 5, respectively), SBP at admission: 2 (2 in group 5), lowest blood pressure during admission: 8 (1, 2, and 5 in group 3, 4, and 5, respectively), PS: 4 (4 in group 5), TP: 8 (2 and 6 in group 3 and 5, respectively), estimated glomerular filtration rate: 1 (1 in group 4), sodium; 2 (1 in group 4 and 5), number of drugs, FRIDs and PIMs: 0

### Medications playing a role in the fall episode

**Fig. 2:**
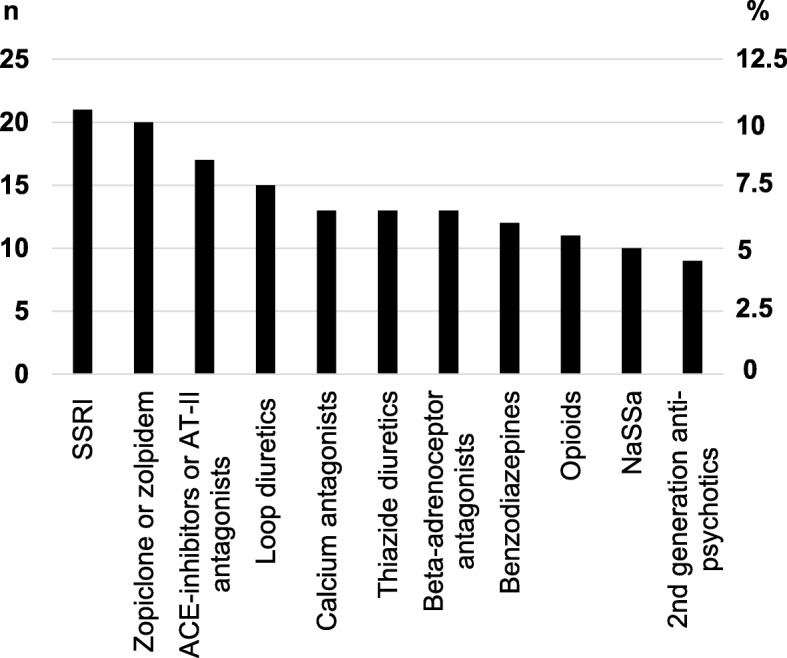
Medications most commonly suspected of contributing to falls. Figure 2. Drug classes most frequently suspected to contribute to the fall in patients with a suspected medication-related fall (group 5, Fig. 1). Left axis shows the crude number of patients in which the drug contribute. Right axis shows the percentage. Abbreviations: SSRI: selective serotonin reuptake inhibitors, ACE: angiotensin-II converting enzyme, AT-II: angiotensin-II receptor, NaSSa: Noradrenergic and specific serotonergic antidepressants

One medication was considered likely to play a role in the fall episode in 16 (19.5%) patients, and more than one medication was considered likely to a role in the fall episode in 66 (80.5%) of the 82 patients with a suspected medication-related fall. All medications suspected to contribute to falls were FRIDs. Psychotropic medications, including antidepressants, benzodiazepines, benzodiazepine-like medications, antipsychotics, antiepileptics and opioids, were suspected of contributing in 68 (83%) patients, whereas antihypertensive medications and diuretics likely played a role in 30 (36.5%) and 29 (35%) of the 82 patients with a suspected medication-related fall, respectively. The medication subclasses likely to have contributed to the fall episodes in more than five patients are shown in Fig. 2.

### PIMs.

PIMs were identified in 141 patients (70.5%). Among patients with a suspected medication-related fall, PIMs were identified in 79 (96%) patients compared to 62 (52%) of the 118 patients without a suspected medication-related fall (*p* < 0.001). In 74 (90%) of the 82 patients with a suspected medication-related fall (37% of the total population), PIMs were suspected to have contributed directly to the fall leading to admission. A single medication was considered both a PIM and a contributor to the fall in 36 (44%) patients, and more than one medication was considered a PIM and contributor to the fall in 38 (46%) of the 82 patients with a suspected medication-related fall. In 42 (51%) of the patients with a suspected medication-related fall, the number of medications found likely to be involved in the fall episode was higher than the number of identified PIMs.

## Discussion

We found that the prevalence of suspected medication-related falls leading to hip a fracture among elderly patients was 41%. Furthermore, we identified at least one of the medications found to contribute to the fall as potentially inappropriate in 90% of patients with a suspected medication-related fall.

The clinical data point to an influence of factors other than medication, e.g., extrinsic sources or chronic or acute illness, in the incident leading to a hip fracture in more than half of the patients. However, the estimated 41% prevalence of suspected medication-related falls suggests that medications are a major risk factor. We have not identified other studies estimating the prevalence of medication-related falls in hip fracture patients, and we were expecting a higher occurrence of suspected medication-related falls due to the frequent use of FRIDs in hip fracture patients [10, 11]. Medications can be considered modifiable risk factors, and we identified an overlap between medications found to contribute to the fall episode and medications identified as PIMs in the majority of the patients with a suspected medication-related fall. Interestingly, the number of medications suspected to be involved in the fall episode was often higher than the number identified as PIMs. Hence, a medication review may reduce the risk of medication-related falls but cannot eliminate it in all at-risk patients, suggesting that the prevalence of potentially *avoidable* medication-related fall-induced hip fractures might be markedly lower than 41%. This suggests that trials exploring the effect of medication reviews either requires a very large number of participants with falls or should be targeted at high risk groups. Our data may guide studies on the latter. In line with this, it has yet to be proven that withdrawal of medications reduces the risk of falls [9, 14, 20]. The randomized study by Boye et al. [9] showed that withdrawal of FRIDs did not alter the fall rate or number of FRIDs after 12 months, and they proposed a lack of compliance with the withdrawal or prescription of new medications as possible explanations [9]. Accordingly, the number of FRIDs may actually increase after a hip fracture, [21] indicating that medication reviews among elderly patients should be a primary prophylactic modality. To select patients in whom to perform a medication review, our data points towards patients prescribed a higher number of medications and FRIDs, as these were the only obvious risk factors that detected those individuals with a suspected medication-related fall. The finding of a higher prevalence of ischaemic heart disease and previous fractures may be explained by an association of these conditions with a higher usage of medications.

We found that the most common FRIDs associated with suspected medication-related falls were psychotropic medications, particularly selective serotonin reuptake inhibitors and benzodiazepines or benzodiazepine-like medications, followed by antihypertensives and diuretics. This can be explained by the known adverse effects of these drugs [6, 8] and their widespread use. Thus, it is important to focus on these medications when performing medication reviews in order to reduce the risk of medication related falls. Nevertheless, thiazides may also have a beneficial effect on bone strength by increasing renal calcium reabsorption [22]. The use of medications with an established bone-demineralizing effect, such as oral corticosteroids, aromatase inhibitors and enzyme-inducing anti-epileptics, was infrequent in our population, suggesting a limited contribution to falls by these medications among hip fracture patients. However, we could not evaluate the lifetime use of medications, and the long-term effect of prior use of such medications cannot be excluded in our study.

This study has several limitations. For one thing, the population is relatively small. The retrospective design implies that we had to rely on data obtained routinely and the possibility of focused examinations and interviews was precluded. The evaluation of clinical data and falls was not blinded to the list of medications, and the evaluations of the role of medications may have varied depending on the observer. On the other hand, the retrospective design [23] allowed us to study an unselected population of consecutive elderly patients with hip fractures, with access to clinical data, laboratory data and a detailed description of the fall episode. The validity of the description of the fall episode in the patient record is strengthened by the fact that this is a designated clinical task for the on-site consultant in geriatrics. Furthermore, the joint expertise of the clinical pharmacologist and the geriatrician in the evaluation of the individual patient records strengthens the evaluations and conclusions in our report. The female preponderance, age, high frequency of comorbidities, widespread use of medications, FRIDs, and PIMs in our patients correspond well to those from other studies of Danish and international cohorts of patients with hip fractures [11, 12, 24–26]. Thus, our population may be a representative sample of elderly patients with hip fracture. Altogether, we consider our result to be a qualified estimate of the prevalence of medication-related falls in elderly hip fracture patients.

## Conclusions

The prevalence of suspected medication-related falls was 41% in elderly patients admitted with a hip fracture. It seems likely that a medication review followed by withdrawal of inappropriate medications could have reduced, though not eliminated, the risk of falling in 74 (37%) of the total population. Still, intervention studies are warranted and the present data may support planning of such studies.

## Supplementary information

**Additional file 1: Table S1.** Medications used at the time of admission

## Data Availability

The datasets generated and analysed during the current study are not publicly available because the Danish Patient Safety Authority has to approve transmission of the data to other researchers in each case. Data are, however, available from the authors upon reasonable request and with permission of the Danish Patient Safety Authority.

## Abbreviations

FRIDS: Fall-risk-increasing drugs
STOPP: Screening Tool of Older Persons’ Prescriptions
PIMs: Potentially inappropriate medications
SD: Standard deviation
ACE: Angiotensin converting enzyme
