# Supplementary material for: Prevalence of medication-related falls in 200 consecutive elderly patients with hip fractures: a cross-sectional study
Source: BMC Geriatr. 2020 Mar 30;20:121. doi: 10.1186/s12877-020-01532-9 (PMC7106700; doi:10.1186/s12877-020-01532-9)
Supplement: Supplementary file 1 — Additional file 1: Table S1. Medications used at the time of admission [file 12877_2020_1532_MOESM1_ESM.docx]

**On-line supplement: Prevalence of medication-related falls in 200 consecutive elderly patients with hip fractures: a cross-sectional study**

**Table S1. Medications used at the time of admission**

|  | **n (%)** | **Category** | **n (%)** | **Class** | **n (%)** | **Medication** | **n (%)** |
| --- | --- | --- | --- | --- | --- | --- | --- |
| **Cardiovascular drugs** | 163 (81.5) | Anti-hypertensives | 116 (58) | ACE-inhibitor or AT-II antagonists | 73 (36.5) | Ramipril | 22(11) |
|  |  |  |  |  |  | Enalapril | 22 (11) |
|  |  |  |  |  |  | Losartan | 26 (13) |
|  |  |  |  |  |  | Other | 5 (2.5) |
|  |  |  |  | Beta-adrenoreceptor antagonist | 59 (29.5) | Metoprolol | 49 (24.5) |
|  |  |  |  |  |  | Other | 10 (5) |
|  |  |  |  | Calcium-antagonist | 47 (23.5) | Amlodipine | 37 (17) |
|  |  |  |  |  |  | Other | 10 (5) |
|  |  | Diuretics | 91 (45.5) | Loop diuretics | 52 (26) | Furosemide | 52 (26) |
|  |  |  |  | Thiazides | 38 (19) | Bendroflumethiazid or Hydrochlorthiazid | 38 (19) |
|  |  |  |  | Other | 12 (6) | Spironolactone | 7 (3.5) |
|  |  |  |  |  |  | Other | 5 (2.5) |
|  |  | Cholesterol lowering | 67 (33.5) | Statins | 67 (33.5) | Simvastatin | 42 (21) |
|  |  |  |  |  |  | Atorvastatin | 21 (10.5) |
|  |  |  |  |  |  | Other | 4 (2) |
|  |  | Thrombocyte-inhibitors | 66 (33) |  |  | Salicylic acid | 46 (23) |
|  |  |  |  |  |  | Clopidogrel | 22 (11) |
|  |  | Anti-coagulants | 40 (20) | Vitamin K antagonists | 20 (10) | Warfarin | 20 (10) |
|  |  |  |  | NOACS | 18(9) | Rivaroxaban | 10 (5) |
|  |  |  |  |  |  | Apixaban | 6 (3) |
|  |  |  |  |  |  | Dabigatran | 2 (1) |
|  |  |  |  | LMWH | 2 | Fragmin | 2 (1) |
|  |  | Anti-arrhythmics | 11 (5.5) |  |  | Digoxin | 7 (3.5) |
|  |  |  |  |  |  | Amiodarone | 2 (1) |
|  |  |  |  | Calcium channel antagonists | 2 (1) | Diltiazem | 2 (1) |
|  |  | Vasodilators | 6 (3) | Nitrates | 6(3) | Glycerolnitrate | 5 (2.5) |
| **Analgesics** | 113 (56.5) | Weak analgesics | 107 (53.5) |  |  | Paracetamol | 105 (52.5) |
|  |  |  |  | NSAIDs | 9 (4.5) | Ibuprofen | 9 (4.5) |
|  |  | Opioids | 37 (18.5) |  |  | Tramadol | 20 (10) |
|  |  |  |  |  |  | Morphine | 11 (5.59 |
|  |  |  |  |  |  | Buprenorphine | 4 (2) |
|  |  |  |  |  |  | Other | 3 (1.5) |
| **Psychotropic medications** | 98 (54) | Anti-depressants | 53 (26.5) | SSRI | 34(17) | Citalopram | 20 (10) |
|  |  |  |  |  |  | Sertralin | 9 (4.5) |
|  |  |  |  |  |  | Escitalopram | 4 (2) |
|  |  |  |  | NaSSA | 17 (8.5) | Mirtazapin | 17 (8.5) |
|  |  |  |  | SNRI | 6 (3) | Duloxetine | 3 (1.5) |
|  |  |  |  |  |  | Venlafaxine | 3(1.5) |
|  |  |  |  | TCA | 4 (2) | Amitriptyline | 2 (1) |
|  |  |  |  |  |  | Other | 2 (1) |
|  |  |  |  | Other | 2 (1) | Agomelatine | 2(1) |
|  |  | Benzo-diazepines | 35 (18) | Benzodiazepine-like drugs | 21 (10.5) | Zopiclone | 14 (7) |
|  |  |  |  |  |  | Zolpidem | 7 (3.5) |
|  |  |  |  | Benzodiazepines | 17 (8.5) | Oxazepam | 4 (2) |
|  |  |  |  |  |  | Diazepam | 3 (1.5) |
|  |  |  |  |  |  | Bromazepam | 3 (1.5) |
|  |  |  |  |  |  | Alprazolam | 3 (1.5) |
|  |  |  |  |  |  | Other | 4 (2) |
|  |  | Antipsychotics | 17 (8.5) | 2nd generation | 12 (6) | Risperidone | 5 (2.5) |
|  |  |  |  |  |  | Olanzapine | 4 (2) |
|  |  |  |  |  |  | Quetiapine | 4 (2) |
|  |  |  |  | 1st generation | 5 (2.5) | Haloperidol, Chlorprothixen, Zuclopenthixol or other | 5 (2.5) |
|  |  | Anti-epileptics | 16 (8) | Other | 11 (5.5) | Carbamazepine | 4 (2) |
|  |  |  |  |  |  | Lamotrigine | 4 (2) |
|  |  |  |  |  |  | Levetirazetam or  Valproate | 3 (1.5) |
|  |  |  |  | Gabapentinoids | 6 (3) | Gabapentin | 3 (1.5) |
|  |  |  |  |  |  | Pregabalin | 3 (1.5) |
|  |  | Dementia | 16 (8) | Acetylcholinesterase-inhibitors | 11 (5.5) | Donepezil | 10 (5) |
|  |  |  |  | NMDA-receptor antagonists | 7 (3.5) | Memantin | 7 (3.5) |
|  |  | Anti-Parkinson’s | 8 (4) | Levodopa |  | Levodopa + decarboxylase inhibitor | 4 (2) |
|  |  |  |  | Dopamine agonists | 3 (1.5) | Pramipexol | 2 (1) |
|  |  | Other | 6 (3) | 1st generation antihistamines | 3 (1.5) | Promethazin | 3 (1.5) |
|  |  |  |  | Other | 3 (1.5) | Melatonin | 3 (1.5) |
| **Gastrointestinal medications** | 130 (65) | Vitamins and minerals | 87 (43.5) |  |  | Vitamin-D* | 87 (43.5) |
|  |  |  |  |  |  | Calcium supplement | 60 (30) |
|  |  |  |  |  |  | Potassium | 47 (23.5) |
|  |  | Laxatives | 66 (33) | Osmotic | 63 (31.5) | Macrogol | 44 (22) |
|  |  |  |  |  |  | Magnesia | 17 (8.5) |
|  |  |  |  |  |  | Lactulose | 5 (2.5) |
|  |  |  |  | Peristalsis-increasing | 15 (7.5) | Bisacodyl | 8 (4) |
|  |  |  |  |  |  | Natriumpicosulfat | 7 (3.5) |
|  |  | Acid lowering agents | 50 (24) | Proton pump inhibitors | 49 (24.5) | Pantoprazole | 33 (16.5) |
|  |  |  |  |  |  | Lanzoprazole | 10 (5) |
|  |  |  |  |  |  | Omeprazole | 6 (3) |
|  |  | Nausea | 4 (2) | Dopamine agonists | 3 (1.5) | Metoclopramid | 3 (1.5) |
| **Endocrine drugs** | 76 (38) | Anti-osteoporotic medications | 38 (19) | Bisphosphonates | 31 (15.5) | Alendronate | 28 (14) |
|  |  |  |  |  |  | Other | 3 (1.5) |
|  |  |  |  | Monoclonal antibodies | 5 (2.5) | Denusomab | 5 (2.5) |
|  |  | Oral Antidiabetics | 16 (8) |  |  | Metformin | 16 (8) |
|  |  |  |  | DDP-4 inhibitors | 3 (1.5) | Sitagliptin | 3(1.5) |
|  |  |  |  | Sulfonylureas | 3 (1.5) | Glimepirid | 3 (1.5) |
|  |  | Insulin | 14 (7) |  |  | Insulin | 14 (7) |
|  |  | Thyroid disorders | 16 (8) | Thyroid hormones | 10 (5) | Levothyroxine | 10 (5) |
|  |  |  |  | Thiouracil derivatives | 8 (4) | Thiamazol | 8 (4) |
|  |  | Glucocorticoids | 5 (2.5) | Synthetic glucocorticoids |  | Prednisolone | 5 (2.5) |
| **Respiratory medications** | 47 (23.5) | Inhaled therapy for COPD or asthma | 37 (18.5) | Short-acting beta-agonists | 29 (14.5) |  |  |
|  |  |  |  | Long-acting beta-agonists | 27(13.5) |  |  |
|  |  |  |  | Inhaled glucocorticoids | 24 (12) |  |  |
|  |  |  |  | Long-acting muscarinic-antagonist | 20 (10) |  |  |
|  |  |  |  | Short-acting muscarinic-antagonists | 3 (1.5) |  |  |
|  |  | Anti-allergic drugs | 10 (5) | Non-sedating antihistamines | 6 (3) | Fexofenadine | 2 (1) |
|  |  |  |  |  |  | Other | 4 (2) |
|  |  |  |  | Nasal glucocorticoid | 3 (1.5) | Fluticasonfuroate | 2(1) |
| **Urogenital medications** | 23 (11.5) | Medications for hypertrophy of the prostate | 13 (6.5) | Alpha-adrenergic blockers | 11 (5.5) |  |  |
|  |  |  |  | 5-alpha reductase inhibitors | 4 (2) | Finasterid | 4 (2) |
|  |  | Urinary antispasmodics | 10 (5) | Anticholinergics | 6 (3) | Tolterodin | 2(1) |
|  |  |  |  |  |  | Fesoterodin | 2 (1) |
|  |  |  |  | Beta-3 adrenoceptor agonists | 5 (2.5) | Mirabegron | 5 (2.5) |
| **Eyes** | 28 (14) | Eye drops | 28(14) | Prostanoids | 10 (5) |  |  |
|  |  |  |  | Beta-adrenoceptor antagonists | 8 (4) | Timolol | 8(4) |
|  |  |  |  | Other | 18 (9) |  |  |
| **Miscellaneous** | 22 (11) | Restless legs | 9 (4.5) | Antimalarials | 9 (4.5) | Quinine | 9 (4.5) |
|  |  | Systemic antibiotics | 7 (2.5) | Beta-lactams | 4 (2) | Penicillin | 2 (1) |
|  |  |  |  |  |  | Amoxicillin + clavulanic acid | 2 (1) |
|  |  |  |  | Other | 2 (1) | Trimethoprim | 2 (1) |
|  |  | Rheumatic disorders | 7 (4.5) | Urate synthesis-inhibitors | 6 (3) | Allopurinol | 6 (3) |
|  |  |  |  | Other | 2 (1) | Colchicine | 2 (1) |
|  |  | Antioestrogens | 2 (1) | Aromatase inhibitors | 2 (1) | Letrozol | 2 (1) |

Table S1: vitamin-D supplement of > or = 20 ug/day. One patient could use several types of drugs. Abbreviations: ACE: angiotensin converting enzyme, AT-II: angiotensin-II receptor, LMWH: low-molecular-weight heparin, NSAIDs: nonsteroidal anti-inflammatory medications, PPI: proton pump inhibitors, SSRI: selective serotonin reuptake inhibitors, SNRI: serotonin and noradrenaline reuptake inhibitors, TCA: tricyclic antidepressants, SABA: short-acting beta-agonists, LAMA+ICS: long-acting beta adrenoceptor-agonists + inhaled corticosteroids, NOAC: non-warfarin oral anticoagulants, NMDA: N-Methyl-D-aspartate, NaSSa: noradrenergic and specific serotonergic antidepressants, DDP-4: dipeptidyl peptidase-4.
